# Prognostic Significance of Plasma Short-Chain Fatty Acid Levels in Assessing Mortality Risk in Patients with Chronic Heart Failure and Sarcopenia

**DOI:** 10.3390/ijms26135984

**Published:** 2025-06-22

**Authors:** Anna V. Sokolova, Dmitrii O. Dragunov, Anastasiya V. Klimova, Yaroslav V. Golubev, Tatiana A. Shmigol, Vadim V. Negrebetsky, Gregory P. Arutyunov

**Affiliations:** 1 Federal State Autonomous Educational Institution of Higher Education ‘N.I. Pirogov Russian National Research Medical University’ of the Ministry of Health of the Russian Federation, 117513 Moscow, Russia; sokolova2211@gmail.com (A.V.S.); klimova_av@rsmu.ru (A.V.K.); tolstoyliterature@mail.ru (Y.V.G.); shmigol_ta@rsmu.ru (T.A.S.); negrebetsky1@rsmu.ru (V.V.N.); arut@ossn.ru (G.P.A.); 2 Research Institute of Health Organization and Medical Management of the Department of Health of Moscow, 115184 Moscow, Russia; 3 City Clinical Hospital No. 3 of the Department of Health of Moscow, 220030 Moscow, Russia

**Keywords:** short-chain fatty acids, hexanoic acid, sarcopenia, chronic heart failure, mortality

## Abstract

Short-chain fatty acids (SCFAs) are microbial metabolites involved in immune regulation, energy metabolism, and intestinal barrier integrity. Among them, the role of hexanoic acid (C6), predominantly derived from dietary sources, remains poorly understood in chronic heart failure (CHF) and sarcopenia. A total of 636 patients with confirmed CHF were screened between 2019 and 2021. Sarcopenia was diagnosed in 114 patients, with 74 meeting the inclusion criteria for analysis. Plasma levels of SCFAs—including butanoic, propanoic, isobutyric, 2- and 3-methylbutanoic, hexanoic, pentanoic, and 4-methylpentanoic acids—were measured using HPLC-MS/MS. Muscle strength, mass, and physical performance were assessed using handgrip dynamometry, bioelectrical impedance analysis, and SPPB, respectively. All patients showed elevated SCFA levels compared to reference values. Butanoic acid levels exceeded reference values by 32.8-fold, propanoic acid by 10.9-fold, and hexanoic acid by 1.09-fold. Patients with plasma hexanoic acid levels above the 50th percentile had a seven-fold increased mortality risk (OR = 7.10; 95% CI: 1.74–28.9; *p* < 0.01). Kaplan–Meier analysis confirmed significantly lower survival in this group (*p* = 0.00051). The mean left ventricular ejection fraction was 41.2 ± 7.5%, and the mean SPPB score was 6.1 ± 1.8, indicating impaired physical performance. Elevated plasma hexanoic acid is associated with poor prognosis in CHF patients with sarcopenia. These findings suggest that C6 may serve as a potential prognostic biomarker and therapeutic target in this population.

## 1. Introduction

Hexanoic acid (also known as caproic acid) is a saturated carboxylic acid with a six-carbon chain and the chemical formula C_6_H_12_O_2_. Within the domain of human physiology, hexanoic acid is classified as a short-chain fatty acid (SCFA), which is produced through the fermentation of dietary fiber by the gut microbiota. SCFAs, including acetate, propionate, and butyrate, are well-known for their beneficial effects on host health, such as modulation of inflammatory responses, maintenance of intestinal barrier integrity, and involvement in energy metabolism. However, the role of hexanoic acid in these physiological processes remains poorly understood.

Evidence suggests that elevated levels of certain SCFAs in plasma may be associated with unfavorable metabolic conditions. For instance, in the study by M. Thing et al. [1], patients with metabolic-associated steatotic liver disease (MASLD) exhibited increased plasma concentrations of propionate, butyrate, and valerate, which may indicate their potential involvement in the onset or progression of the disease.

In the context of chronic heart failure (CHF) and sarcopenia, data regarding the effects of hexanoic acid are scarce. Nonetheless, it is well established that dysregulation of short-chain fatty acid (SCFA) profiles can influence inflammatory signaling and muscle metabolism. This is of particular relevance for patients suffering from congestive heart failure (CHF) and sarcopenia. Elevated levels of specific SCFAs have been demonstrated to promote systemic inflammation, disrupt energy homeostasis, and accelerate skeletal muscle degradation. Collectively, these factors contribute to poor clinical outcomes in individuals with CHF.

It should be emphasized that even hexanoic acid, despite its structural differences from more traditional SCFAs (C2–C5), is also synthesized with the participation of the gut microbiota and reflects the metabolic activity of microorganisms in the intestinal lumen [2]. It can influence the expression of pro-inflammatory cytokines, activate TLR2/PPARγ receptors, and disrupt the balance between HIF-1α and HIF-2α, which is associated with impaired systemic metabolism, inflammation, and muscle catabolism in patients with chronic heart failure (CHF) [3,4]. Thus, SCFAs represent a key component of the “gut–microbiota–metabolites” axis, and their role in the pathogenesis of CHF requires further investigation.

Therefore, evaluating plasma levels of hexanoic acid in patients with CHF and sarcopenia is of scientific interest to better understand its potential role in cardiovascular complications and muscle dysfunction. Further research is warranted to elucidate the clinical significance of hexanoic acid as a biomarker and its potential as a therapeutic target in this patient population.

## 2. Results

A total of 636 patients with a confirmed diagnosis of chronic heart failure (CHF) were screened. In 63 patients, assessment of body composition using bioelectrical impedance analysis was not feasible due to the presence of implanted electronic devices (e.g., pacemakers). Sarcopenia was identified in 114 patients; however, only 74 of them met the inclusion and exclusion criteria (Table 1) and were included in the final analysis.

The follow-up period was 1.5 years, during which plasma levels of various short-chain fatty acids (SCFAs), including hexanoic acid, were analyzed in relation to adverse cardiovascular events and all-cause mortality.

The mean age of participants was 68.3 ± 5.7 years, with 58% being male. A high prevalence of comorbid conditions commonly observed in CHF and sarcopenia patients was documented:

- Arterial hypertension—81%;
- Type 2 diabetes mellitus—36%;
- Chronic kidney disease—42%;
- Coronary artery disease—67%;
- Atrial fibrillation—28%;
- Post-infarction cardiosclerosis—32%.

All patients included in the study were hospitalized at City Clinical Hospital No. 4 of the Moscow City Healthcare System and received standard hospital meals. The hospital uses a standardized menu that complies with dietary recommendations for patients with chronic heart failure (Pevzner diet No. 10). Patients with concomitant diabetes mellitus (36% of the sample) received a modified diet adapted in terms of carbohydrate content, in accordance with therapeutic diet No. 9 requirements.

Despite these different dietary approaches, the daily calorie content of the diets remained comparable from a physiological perspective. In addition, the basal metabolic rate (BMR), which reflects individual energy requirements, was assessed in all patients using bioimpedance analysis. The average BMR was 1486 ± 173 kcal/day, indicating that the caloric load and nutritional structure were comparable among the study participants.

Cardiological examination enabled patients to be categorized according to the echocardiographic approach to CHF phenotypes. Among the seventy-four patients included, the diagnoses were as follows:

Twelve (20%) were diagnosed with heart failure with reduced ejection fraction (HFrEF, LVEF < 40%);

Fourteen (23%) had heart failure with a moderately reduced ejection fraction (HFmrEF, LVEF 40–49%), and thirty-five (57%) had heart failure with a preserved ejection fraction (HFpEF, LVEF ≥ 50%).

The mean left ventricular ejection fraction was 49% ± 13%, the left ventricular mass index (LVMI) was 101 g/m^2^ ± 46, the end-diastolic volume (EDV) was 115 mL ± 36, the end-systolic volume (ESV) was 62 mL ± 36, the end-diastolic dimension (EDD) was 5.02 ± 0.59 cm and the end-systolic left ventricular (LV) dimension (ESD) was 5.8 ± 10.2 cm. The mean pulmonary artery pressure (MPAP) was 38 ± 10 mmHg, and the left atrial volume was 76 ± 39 mL.

The mean B-type natriuretic peptide (BNP) concentration was 1327 pg/mL (885–2340 pg/mL), with 51 patients (75%) having levels above 2340 pg/mL.

Sarcopenia was diagnosed in accordance with EWGSOP2 criteria, including assessments of muscle strength, muscle mass, and physical performance.

The mean handgrip strength was 18.5 ± 3.8 kg in men and 14.2 ± 2.9 kg in women, which was significantly below the reference values.

Total skeletal muscle mass, as measured by bioelectrical impedance analysis, averaged 27.8 ± 3.9 kg, indicating marked muscle loss.

Assessment using the Short Physical Performance Battery (SPPB) yielded a mean score of 6.1 ± 1.8 points, reflecting substantial impairments in physical function and a high risk of falls.

The hematological parameters reflecting the peripheral blood count in the study participants were, on average, within the reference range (Table 2).

Statistical analysis revealed a moderate but significant positive correlation between hexanoic acid levels and hs-CRP (r = 0.48; *p* = 0.048). This suggests a potential association between this short-chain fatty acid and systemic inflammation in patients with chronic heart failure (CHF) and sarcopenia. At the same time, no significant correlations were found between hexanoic acid levels and other inflammatory indices: NLR: r = 0.01, *p* = 0.97; SII: r = −0.06, *p* = 0.82; PLR: r = 0.09, *p* = 0.62; MLR: r = 0.15, *p* = 0.40; LMR: r = −0.15, *p* = 0.40.

One of the key objectives of the study was to evaluate plasma concentrations of SCFAs. Measured analytes included acetic acid, propanoic acid, butanoic acid, isobutyric acid, pentanoic acid, 3-methylbutanoic acid, 2-methylbutanoic acid, 4-methylpentanoic acid, and hexanoic acid.

All patients included in the final analysis exhibited elevated plasma SCFA concentrations exceeding reference values (Table 3). The most pronounced increase was observed in butanoic acid, with a 32.8-fold elevation above the reference range. Propanoic acid levels exceeded normal values by a factor of 10.9.

Elevations were also noted for other SCFAs: isobutyric acid (2.82-fold), 2-methylbutanoic acid (1.11-fold), 3-methylbutanoic acid (1.12-fold), hexanoic acid (1.09-fold), 4-methylpentanoic acid (1.20-fold), and pentanoic acid (1.33-fold).

**Figure 1:**
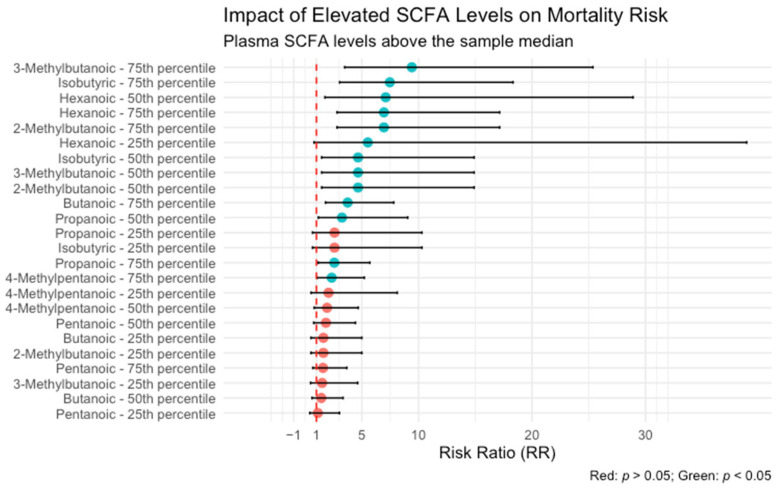
Risk of mortality according to plasma levels of short-chain fatty acids (SCFAs). Patients with hexanoic acid concentrations above the 50th percentile demonstrated a significantly increased risk of death (OR = 7.10; 95% CI: 1.74–28.9).

Analysis of the association between plasma SCFA levels and patient outcomes revealed that hexanoic acid exerted the most pronounced influence on mortality risk. As illustrated in Figure 1, plasma concentrations of hexanoic acid above the 50th percentile were associated with a seven-fold increase in the risk of death (odds ratio [OR] = 7.10; 95% confidence interval [CI]: 1.74–28.9).

**Figure 2:**
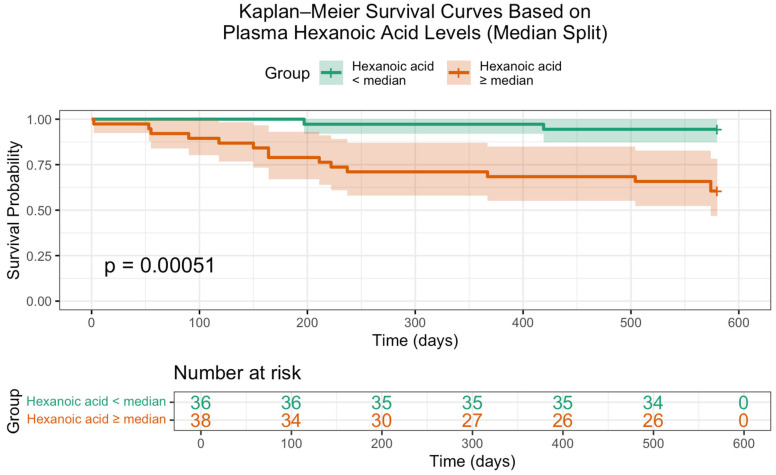
Kaplan–Meier survival curves stratified by serum hexanoic acid levels relative to the median value. Patients with plasma hexanoic acid levels below the median demonstrated significantly improved survival compared to those with higher levels (log-rank *p* = 0.00051).

Additionally, Kaplan–Meier survival analysis (Figure 2) confirmed a statistically significant difference in overall survival based on hexanoic acid levels. Patients with lower concentrations of this metabolite exhibited markedly improved survival probabilities (log-rank *p* = 0.00051), underscoring its potential prognostic value.

## 3. Discussion

Hexanoic acid (C6) is predominantly derived from dietary sources rather than being produced by gut microbiota. The substance is present in the form of triacylglycerol in coconut and palm oils, and is metabolized by the human body as an energy substrate [5,6]. Unlike acetate to valerate (C2–C5), hexanoic acid is generated through β-the oxidation of long-chain fatty acids in the liver [7,8]. Structurally, C2–C5 and C6 differ by only a few carbon atoms, yet studies have demonstrated considerable differences in their immunological properties.

It is well established that C2–C5 exhibit anti-inflammatory properties [9], particularly through the maintenance of intestinal epithelial integrity by mitigating the inflammatory microenvironment at the mucosal interface [7]. This effect is associated with the decreased activity of nuclear factor κB (NF-κB) and an increased release of prostaglandin E2 [10,11,12]. The literature reports that C2–C5 reduce the production of pro-inflammatory cytokines such as interleukin-1β (IL-1β), IL-6, and tumor necrosis factor-alpha (TNF-α) [13]. These effects are best characterized for butyric acid (C4), particularly in the presence of lipopolysaccharides acting via toll-like receptor 4 (TLR4). One hypothesis posits that TLR4, a homodimer signaling through adaptor proteins MyD88/MAL and TRIF/TRAM, is evolutionarily conserved and plays a sentinel role in the regulation of core physiological responses [14]. The microbiota-derived production of C2–C5, with their pleiotropic functions in epithelial maintenance and regulation of cellular proliferation, aligns with the evolutionarily conserved pattern of TLR4 signaling. Specifically, C4-induced anti-inflammatory effects have been shown to act through TLR4-mediated pathways, suppressing the expression of the adaptor protein TRAF6 [15,16].

In contrast, hexanoic acid (C6) has been linked to pro-inflammatory effects, not only through the enhanced production of pro-inflammatory cytokines but also via the suppression of IL-10 production through the TLR2 signaling pathway [13]. Typically, TLR2 functions as a heterodimer. From an evolutionary perspective, the hypothesis is that TLR2 demonstrates plasticity in its ability to respond to diverse and dynamic environmental ligands and pathogens. These include pathogen-associated molecular patterns (PAMPs), microbial-associated molecular patterns (MAMPs), and damage-associated molecular patterns (DAMPs) [17].

In the present study, elevated plasma levels of hexanoic acid above the 50th percentile were found to be associated with a seven-fold increase in mortality risk. C6 stimulation has been linked to increased transcription of GPR84 and PPARγ, two receptors thought to mediate C6’s pro-inflammatory actions and further promote IL-6 and IL-1β expression [18]. It is evident that peroxisome proliferator-activated receptor gamma (PPARγ) exerts a significant influence on both insulin sensitivity and adipogenesis. Statistical analysis revealed a moderate yet significant positive correlation between hexanoic acid levels and hs-CRP (r = 0.48; *p* = 0.048), suggesting a potential association between this short-chain fatty acid and systemic inflammation in patients with chronic heart failure (CHF) and sarcopenia. However, no significant correlations were found between hexanoic acid levels and other inflammatory indices: NLR (r = 0.01, *p* = 0.97), SII (r = −0.06, *p* = 0.82), PLR (r = 0.09, *p* = 0.62), MLR (r = 0.15, *p* = 0.40), or LMR (r = −0.15, *p* = 0.40).

Another immunoregulatory consequence of C6 exposure is the altered ratio of HIF-1α to HIF-2α. These HIF-α subunits have been identified as transcription factors that regulate cellular metabolism and angiogenesis in response to hypoxic and inflammatory stress. It is evident that HIF-1α exerts a predominant role in the regulation of genes associated with glycolysis and NADPH regeneration under hypoxic conditions [19]. Rigorous investigation has demonstrated that inflammation instigated by C6 results in the suppression of HIF-1α transcription and the preferential induction of HIF-2α. Conversely, exposure to C4 has been demonstrated to exert a negligible influence on HIF-1α expression. Nevertheless, existing data suggest that C4 may stabilize HIF-1α under hypoxic conditions, for example in the intestinal epithelium [14]. HIF-2α is believed to regulate a broader spectrum of target genes, including those involved in inflammatory responses and lipid metabolism. These findings indicate that the altered HIF-1α/HIF-2α activation may represent a compensatory response to enhanced inflammation induced by hexanoic acid [13,17,19,20].

These mechanistic insights into hexanoic acid (C6)-induced inflammation provide a molecular backdrop for understanding clinical outcomes in frail elderly populations. Notably, B-type natriuretic peptide (BNP), a cardiovascular biomarker, has similarly been shown to reflect systemic vulnerability. In a landmark study, Di Marca et al. (2018) [21] demonstrated that BNP levels greater than 600 pg/mL, in conjunction with chronic kidney disease, malnutrition, advanced age, and immobilization syndrome, independently predicted 30-day post-discharge mortality—even among elderly patients admitted for non–heart failure diagnoses. Furthermore, CKD, elevated white blood cell count, immobilization syndrome, and age were also independent predictors of in-hospital mortality in their cohort. These findings emphasize BNP’s role as a global prognostic marker of frailty and multisystem stress in older adults [21].

In our cohort, the mean BNP level was 1327 pg/mL (IQR: 885–2340), and 75% of patients had values exceeding 2340 pg/mL, underscoring the severity of underlying cardiac dysfunction. Although our study focused primarily on long-term mortality risk over a 1.5-year follow-up, these data support the notion that elevated BNP may reflect not only cardiac strain but also multisystem vulnerability associated with sarcopenia, chronic inflammation, and comorbidity burden. Further research is warranted to investigate the differential roles of BNP and related covariates in predicting short-term versus long-term mortality in this patient population.

## 4. Materials and Methods

This study was conducted at City Clinical Hospital No. 4, Moscow Healthcare Department (GKB No. 4 DZM), between September 2019 and December 2021. A total of 636 patients with a confirmed diagnosis of chronic heart failure (CHF) were screened. In 63 patients, body composition analysis via bioelectrical impedance was not feasible due to the presence of implanted cardiac pacemakers. Sarcopenia was identified in 114 patients; however, only 74 met the inclusion and exclusion criteria (Table 1) and were included in the final analysis.

Sarcopenia was diagnosed in accordance with the recommendations of the European Working Group on Sarcopenia in Older People 2 (EWGSOP2) [22]. Skeletal muscle mass was assessed using a bioelectrical impedance analyzer (ABC-02, MEDASS, Moscow, Russia). Handgrip strength was measured using a manual dynamometer (DK-25). Physical performance was evaluated using the Short Physical Performance Battery (SPPB).

All patients underwent comprehensive clinical assessment, including blood sampling to determine plasma levels of short-chain fatty acids (SCFAs), specifically butanoic acid, propanoic acid, isobutyric acid, 2-methylbutanoic acid, 3-methylbutanoic acid, hexanoic acid, pentanoic acid, and 4-methylpentanoic acid. Quantification of SCFAs was performed using high-performance liquid chromatography coupled with mass spectrometry (HPLC-MS/MS).

Reference values for SCFAs were established based on the monitored MRM transitions of their derivatized forms:

- Propanoic acid (C3): 180.05 → 91.05 ng/mL;
- Butanoic acid (C4) and isobutyric acid (iC4): 194.10 → 91.05 ng/mL;
- Pentanoic acid (C5), 2-methylbutanoic acid (αC5), and 3-methylbutanoic acid (βC5): 208.15 → 91.05 ng/mL;
- 4-Methylpentanoic acid (iC6) and hexanoic acid (C6): 222.10 → 91.05 ng/mL.

### Statistical Analysis

Statistical analysis was performed using R language (version 4.3.1) and RStudio software (version 2024.12.0.467, http://www.posit.co/, Posit, Boston, MA, USA). Data distribution was assessed using the Shapiro–Wilk test. Continuous variables were presented as mean ± standard deviation (SD) for normally distributed data or as median and interquartile range (IQR) for non-normally distributed variables. Categorical variables were summarized as frequencies and percentages.

Group comparisons were conducted using Student’s *t*-test or the Mann–Whitney U test for continuous variables, and the chi-squared test or Fisher’s exact test for categorical variables, as appropriate. Paired data before and after the intervention were analyzed using the Wilcoxon signed-rank test or paired *t*-test, depending on normality.

The association between plasma SCFA concentrations and mortality risk was evaluated using logistic regression analysis. Odds ratios (ORs) and 95% confidence intervals (CIs) were calculated. Survival analysis was performed using Kaplan–Meier curves, and differences between groups were assessed with the log-rank test.

A two-tailed *p*-value of less than 0.05 was considered statistically significant.

## 5. Conclusions

In patients with chronic heart failure (CHF) and sarcopenia, plasma levels of short-chain fatty acids (SCFAs) were significantly elevated compared to reference values. The most pronounced increases were observed for butanoic acid (32.8-fold) and propanoic acid (10.9-fold), suggesting impaired intestinal barrier function and altered gut microbiota composition. Elevated concentrations of other SCFAs, including isobutyric, methylbutanoic, and hexanoic acids, also indicated marked metabolic disturbances associated with chronic inflammation. Hexanoic acid demonstrated the highest prognostic significance: plasma levels above the 50th percentile were associated with a seven-fold increase in mortality risk, and Kaplan–Meier survival analysis showed a significant reduction in overall survival in patients with elevated levels of this metabolite (*p* = 0.00051). These findings highlight the potential value of SCFA profiling as a biomarker of adverse prognosis in patients with CHF and sarcopenia.

### Study Limitations

The present study has several limitations. Firstly, it was conducted at a single center, which may restrict the applicability of the findings to a wider population of patients with chronic heart failure (CHF) and sarcopenia. Secondly, while the sample size was sufficient for the initial analysis, the total number of patients and recorded outcomes was relatively small, reducing the statistical power for subgroup analysis and multivariable modeling. Thirdly, plasma concentrations of short-chain fatty acids (SCFAs) are not routinely evaluated in CHF patients and their reference values have not yet been standardized in clinical practice. The method used to quantify SCFAs requires specialized equipment and expertise, which limits reproducibility and practical implementation. To confirm the prognostic value of individual SCFAs and clarify their pathophysiological role in CHF and sarcopenia, multicentre prospective studies with larger sample sizes are needed. A key limitation of this study is the absence of analysis of the composition of the gut microbiota. Although short-chain fatty acids (SCFAs), including hexanoic acid, are known to be microbial metabolites, investigating the intestinal microbiome was beyond the scope of our research design and therefore not performed. This restricts our ability to link the observed alterations in SCFA levels to specific microbial taxa or patterns of dysbiosis. Further research incorporating microbiota profiling (e.g., 16S rRNA gene sequencing or metagenomics) is required to elucidate the gut–heart–muscle axis in chronic heart failure and sarcopenia.

## Figures and Tables

**Table 1:** Inclusion/exclusion criteria for the study.

Inclusion Criteria	Exclusion Criteria
Age ≥ 60 years	Presence of electronic medical devices
Confirmed diagnosis of chronic heart failure (CHF)	Any severe, decompensated, or unstable somatic diseases or conditions that, in the opinion of the researcher, threaten the life of the patient or worsen the prognosis of the disease (including anemia, autoimmune, endocrine, oncological diseases, hepatitis, connective tissue diseases, etc.).
Confirmed diagnosis of sarcopenia	Severe congenital or acquired musculoskeletal disorders
Signed informed consent for participation in the clinical study	Presence of concomitant neurosurgical or neurological pathology
	Abuse of alcohol, drugs, narcotics
	Mental illness, incapacity
	Glomerular filtration rate < 45 mL/min

**Table 2:** Summary of peripheral blood parameters and calculated systemic inflammatory markers.

Parameter	Mean ± SD / Median (IQR)	Units
White blood cells (WBC)	6.79 ± 1.89	×10^9^/L
–Lymphocytes (absolute count)	1.88 ± 0.74	×10^9^/L
–Neutrophils (absolute count)	4.15 ± 1.21	×10^9^/L
–Monocytes (absolute count)	0.60 ± 1.03	×10^9^/L
Platelets (PLT)	225 ± 72	×10^9^/L
High-sensitivity C-reactive protein (hs-CRP)	5.8 ± 2.4	mg/L
Neutrophil-to-lymphocyte ratio (NLR)	1.85 (1.50–2.64)	—
Systemic immune-inflammation index (SII)	419 (288–634)	—
Platelet-to-lymphocyte ratio (PLR)	121 (96–163)	—
Monocyte-to-lymphocyte ratio (MLR)	0.27 (0.21–0.33)	—
Lymphocyte-to-monocyte ratio (LMR)	3.72 (3.00–4.68)	—

**Table 3:** Plasma levels of short-chain fatty acids (SCFAs) in the study population. Values are presented in ng/mL.

SCFA	Minimum	5th Percentile	25th Percentile	Median	75th Percentile	95th Percentile	Maximum
Butanoic acid	6370	7860.5	9752.5	12,300	14,500	22,735	45,000
Propanoic acid	1980	2113.0	2815.0	3745	6110.0	8419.5	17,900
Isobutyric acid	549	606.2	937.8	1860	10,800	18,085	76,205
2-Methylbutanoic acid	197	231.9	419.5	1655	7947.5	14,090	27,000
3-Methylbutanoic acid	93.5	138.6	234.2	638.5	12,725	23,595	29,000
Hexanoic acid	206	243.1	455.0	622.5	1285.0	2659.0	4060
Pentanoic acid	105	203.9	278.8	376.5	491.2	832.0	1253
4-Methylpentanoic acid	52.7	58.8	97.5	114.6	145.4	267.6	390

## Data Availability

The data supporting the findings of this study are available from the corresponding author upon reasonable request. Data are not publicly available due to privacy and ethical restrictions.
